# Supplementary material for: Chiral superconductivity in UTe2 probed by anisotropic low-energy excitations
Source: Nat Commun. 2023 May 23;14:2966. doi: 10.1038/s41467-023-38688-y (PMC10205722; doi:10.1038/s41467-023-38688-y)
Supplement: Supplementary file 1 — Supplementary Information [file 41467_2023_38688_MOESM1_ESM.pdf]

Supplementary Information

I. POSITIONS OF POINT NODES IN CHIRAL SPIN-TRIPLET SUPERCONDUCTING STATES

Here, we discuss the positions of point nodes in chiral superconducting states whose order parameters consist of two components represented by two odd-parity irreducible representations (IRs). First, we consider the $B_{3u} + iB_{1u}$ state. From the basis functions summarized in Table I in the main text, the vector order parameter $\mathbf{d}$ is written by

$$\mathbf{d} = \mathbf{d}_{B3u} + i\mathbf{d}_{B1u} = \begin{pmatrix} c_1 k_x k_y k_z \\ c_2 k_z \\ c_3 k_y \end{pmatrix} + i \begin{pmatrix} c_4 k_y \\ c_5 k_x \\ c_6 k_x k_y k_z \end{pmatrix}, \quad \text{(S1)}$$

where c_1 to c_6 are real coefficients. We note that higher-order terms are neglected, which do not change our discussions below as long as considering a spherical Fermi surface (FS) around the Γ point. In the chiral spin-triplet state, the positions of point nodes in the order parameter $\mathbf{d} = \mathbf{d}_1 + i\mathbf{d}_2$ are derived by the two conditions [S1]

$$\begin{cases} \mathbf{d}_1 \cdot \mathbf{d}_2 = 0 & \text{(S2)} \\ |\mathbf{d}_1| = |\mathbf{d}_2|. & \text{(S3)} \end{cases}$$

By applying the above conditions to the order parameter in Eq. S1, the nodes appear at

$$\begin{cases} k_z = 0 \\ (c_3^2 - c_4^2)k_y^2 = c_5^2 k_x^2 \end{cases} \quad \text{or} \quad \begin{cases} k_x = 0 \\ (c_4^2 - c_3^2)k_y^2 = c_2^2 k_z^2 \end{cases}. \quad \text{(S4)}$$

When $|c_3| > |c_4|$ ($|c_3| < |c_4|$), the first (second) condition induces point nodes, and, with the spherical FS, four point nodes appear on the $k_z = 0$ ($k_x = 0$) plane. Furthermore, qualitatively speaking, when $|\mathbf{d}_{B1u}|$ is much smaller than $|\mathbf{d}_{B3u}|$ ($|c_3| \gg |c_4|, |c_5|$), point nodes appear near the k_x-axis (Fig. 1c), and when $|\mathbf{d}_{B1u}| \approx |\mathbf{d}_{B3u}|$ ($|c_3| \approx |c_4|$), point nodes appear near the k_y-axis.

Next, we consider the $B_{3u} + iA_u$ state. The order parameter $\mathbf{d}$ can be written as

$$\mathbf{d} = \mathbf{d}_{B3u} + i\mathbf{d}_{Au} = \begin{pmatrix} c_1 k_x k_y k_z \\ c_2 k_z \\ c_3 k_y \end{pmatrix} + i \begin{pmatrix} c_4 k_x \\ c_5 k_y \\ c_6 k_z \end{pmatrix}. \quad \text{(S5)}$$

By applying Eqs. S2 and S3 to Eq. S5, we obtain the conditions

$$\begin{cases} k_y = 0 \\ (c_2^2 - c_6^2)k_z^2 = c_4^2 k_x^2 \end{cases} \quad \text{and} \quad \begin{cases} k_z = 0 \\ (c_3^2 - c_5^2)k_y^2 = c_4^2 k_x^2 \end{cases}. \tag{S6}$$

In this case, several nodal structures become possible. First, when $|c_2| > |c_6|$ and $|c_3| > |c_5|$, four point nodes appear on both $k_y = 0$ and $k_z = 0$ planes, and thus eight point nodes appear in total on the spherical FS. Next, when $|c_2| > |c_6|$ and $|c_3| < |c_5|$ ($|c_2| < |c_6|$ and $|c_3| > |c_5|$), four point nodes appear on the $k_y = 0$ ($k_z = 0$) plane. Finally, when $|c_2| < |c_6|$ and $|c_3| < |c_5|$, the superconducting state is fully gapped. Especially, when $|\mathbf{d}_{Au}|$ is much smaller than $|\mathbf{d}_{B3u}|$ ($|c_2| \gg |c_4|, |c_6|$ and $|c_3| \gg |c_4|, |c_5|$), point nodes are near the k_x-axis (Fig. 1d), and when $|\mathbf{d}_{Au}| \approx |\mathbf{d}_{B3u}|$ ($|c_2| \approx |c_6|$ and $|c_3| \approx |c_5|$), point nodes may appear near the k_y- and k_z-axes (Fig. 1e).

II. GAP STRUCTURES WITH TWO ODD-PARITY ORDER PARAMETERS PRESERVING TIME-REVERSAL SYMMETRY

In this section, we discuss the gap structures of the order parameter $\mathbf{d} = \mathbf{d}_1 + \mathbf{d}_2$ preserving time-reversal symmetry, where $\mathbf{d}_1$ and $\mathbf{d}_2$ belong to different IRs. When $\mathbf{d}_1$ and $\mathbf{d}_2$ are represented by B_{3u} and B_{1u} symmetries, respectively, $\mathbf{d}$ can be written as

$$\mathbf{d} = \mathbf{d}_{B3u} + \mathbf{d}_{B1u} = \begin{pmatrix} c_1 k_x k_y k_z + c_4 k_y \\ c_2 k_z + c_5 k_x \\ c_3 k_y + c_6 k_x k_y k_z \end{pmatrix}. \tag{S7}$$

In this case, the positions of nodes are determined from the condition $|\mathbf{d}| = 0$. Therefore, point nodes satisfy the relations

$$\begin{cases} k_y = 0 \\ c_2 k_z = -c_5 k_x \end{cases}, \tag{S8}$$

meaning that two point nodes appear on the $k_y = 0$ plane but off the high-symmetry axes (Fig. S1c). As mentioned in the main text, because the original point nodes of the B_{3u} state is not split by adding the B_{1u} component, the $B_{3u} + B_{1u}$ state cannot account for our experimental results.

In the $B_{3u} + A_u$ state, $\mathbf{d}$ can be represented by

$$\mathbf{d} = \mathbf{d}_{B3u} + \mathbf{d}_{Au} = \begin{pmatrix} c_1 k_x k_y k_z + c_4 k_x \\ c_2 k_z + c_5 k_y \\ c_3 k_y + c_6 k_z \end{pmatrix}. \tag{S9}$$

For the emergence of the point nodes satisfying $|\mathbf{d}| = 0$, the coefficients need to satisfy a specific condition $c_2/c_5 = c_3/c_6$. When this condition is valid, the nodes appear in the points represented by

$$\begin{cases} k_x = 0 \\ c_2 k_z = -c_5 k_y \end{cases}, \tag{S10}$$

and non-split nodes are located on the $k_x = 0$ plane. Therefore, a fine-tuning of the coefficients is required for the emergence of nodes, and even if the coefficients satisfy the specific condition, the $B_{3u} + A_u$ state is inconsistent with our experimental results.

III. POSSIBILITY OF A GAP STRUCTURE WITH LINE NODES

The even-parity IRs of the point group of D_{2h} are listed in Table S1. In the A_{1g} state, a fully-gapped state is realized, which is clearly inconsistent with our experimental results. On the other hand, the B_{1g}, B_{2g}, and B_{3g} states lead to gap structures with line nodes. As mentioned in the main text, a line nodal structure can induce the exponent value $1 \leq n \leq 2$ of the power-law fitting $\Delta\lambda \propto T^n$ because of nonmagnetic impurity scatterings [S2], quantum criticality [S3, S4], and non-local effects [S5]. In the case of UTe_2, although ferromagnetic quantum criticality is expected [S6], the mass renormalization cannot account for the experimental exponents $n_a \approx n_b \approx 2$. Besides, the non-local effects are expected to be too weak due to $\xi/\lambda(0) \sim 0.01$. As for the impurity effect, we stress that crystal #A1 is picked up from the same batch with the sample showing the residual resistivity ratio (RRR) of 1,000 in Ref. [S7]. Indeed, the observed $T_c = 2.1$ K is the highest value ever reported, and from the skin depth analyses, we can estimate the RRR of crystal #A1 as large as 200. Furthermore, we can exclude the presence of line nodes more quantitatively from the C_e/T data. The T dependence of C_e/T in a disordered line nodal gap structure has been calculated in Ref. [S8]. In this previous paper, it has been shown that C_e/T at low temperatures is

always larger than 10 % of the normal state value (γ_{n}) when $\Gamma_{\mathrm{N}} = 0.01 k_{\mathrm{B}} T_{\mathrm{c0}}$, where Γ_{N} is the normal-state scattering time and T_{c0} is the superconducting transition temperature in the clean limit. These calculations contrast to our experimental data with the minimum value of $C_{\mathrm{e}}/T \approx 0.08\gamma_{\mathrm{n}}$ (see Fig. 2e). Therefore, assuming a line-nodal gap structure, we conclude $\Gamma_{\mathrm{N}} < 0.01 k_{\mathrm{B}} T_{\mathrm{c0}}$ in crystal #A1. Then we examine the correspondence between the $\Gamma_{\mathrm{N}}/k_{\mathrm{B}}T_{\mathrm{c0}}$ value and the exponent values obtained from the temperature dependence of the superfluid density. Figure S2a shows the normalized superfluid density in disordered d-wave superconductors with various $\Gamma_{\mathrm{N}}/k_{\mathrm{B}}T_{\mathrm{c0}}$ values. Figure S2b depicts the $\Gamma_{\mathrm{N}}/k_{\mathrm{B}}T_{\mathrm{c0}}$ dependence of the exponent values obtained from the power-law fitting to the superfluid density data. From these calculations, we can find that the estimated $\Gamma_{\mathrm{N}} < 0.01 k_{\mathrm{B}} T_{\mathrm{c0}}$ is too small to change the low-temperature $\Delta\lambda(T)$ behavior from T to T^2 dependence. Thus, we can consider that crystal #A1 is in the clean limit, and the all n_i values near 2 in #A1 data strongly indicate that the gap structure in UTe_2 has point nodes. Furthermore, we found a systematic trend in the evolution of the n_c value, that is the increase of the n_c value aproaching 2 as T_{c} increases. This is clealy an opposite trend expected from the impurity effect on line-nodal superconductors as discussed in the section IX.

We note that, in the presence of point nodes in the gap structure, the impurity scattering can affect only the amplitude of the low-energy excitations, but does not change the exponent value of $\Delta\lambda(T) \propto T^2$ [S9, S10]. Moreover, in the case of UTe_2, several physical properties, such as Knight shift, upper critical field, and reentrant superconductivity, strongly suggest the spin-triplet superconducting state. Considering that the spin-orbit interactions are important in UTe_2 [S11], the spin-triplet state excludes the possibility of the presence of line nodes [S12] (see Table I in the main text).

Next, we discuss mixed-parity states consisting of even-parity order parameter $\psi(\mathbf{k})$ and odd-parity order parameter $\mathbf{d}(\mathbf{k})$. We note that the mixed-parity state in UTe_2 requires the inversion symmetry breaking which is proposed in Ref. [S13] under hydrostatic pressure. When the time-reversal symmetry is broken, because the gap size can be written as $\Delta_{\mathrm{gap}}(\mathbf{k}) = \sqrt{|\psi(\mathbf{k})|^2 + |\mathbf{d}(\mathbf{k})|^2}$, the superconducting state is fully-gapped or has point nodes on the high-symmetry axes. These states are inconsistent with our experimental results.

When the time-reversal symmetry is preserved, on the other hand, the spin split FSs have a different gap size $\Delta^{\pm}_{\mathrm{gap}}(\mathbf{k}) = \psi(\mathbf{k}) \pm |\mathbf{d}(\mathbf{k})|$, leading to various gap structures. In this study, we focus on the mixed-parity states of the B_{1u} or B_{3u} and A_g states, the latter of

which is indeed proposed in Ref. [S13]. When the small A_g component is admixed with the B_{3u} component, the original point nodes on the k_x-axis spread into the nodal rings around the k_x-axis (Fig. S3b). However, such a gap structure with line nodes is inconsistent with our experiments as explained at the beginning of this section.

IV. NON-UNITARY PAIRING STATES

Non-unitary pairing states are defined by $\mathbf{q}(\mathbf{k}) \equiv i\mathbf{d}(\mathbf{k})\times\mathbf{d}^*(\mathbf{k}) \neq 0$ [S14]. In the $B_{3u}+iA_u$ state, $\mathbf{q}(\mathbf{k})$ can be written as

$$\mathbf{q}(\mathbf{k}) = i\mathbf{d}(\mathbf{k})\times\mathbf{d}^*(\mathbf{k}) = -2i\mathbf{d}_{B3u}(\mathbf{k})\times\mathbf{d}_{Au}(\mathbf{k}) = -2i\begin{pmatrix} c_2c_6k_z^2 - c_3c_5k_y^2 \\ k_xk_y(c_3c_4 - c_1c_6k_z^2) \\ k_zk_x(-c_2c_4 + c_1c_5k_y^2) \end{pmatrix}. \quad \text{(S11)}$$

Therefore, the $B_{3u}+iA_u$ state is non-unitary. Physically, this $\mathbf{q}(\mathbf{k})$ corresponds to a spin moment of a Cooper pair, and in the $B_{3u}+iA_u$ case, the average of $\mathbf{q}(\mathbf{k})$ in the FSs $\langle\mathbf{q}(\mathbf{k})\rangle_{\mathrm{FS}}$ is parallel to $\hat{x}$ which is the magnetic easy axis in the normal state [S6]. Usually, the non-unitary state is energetically unstable except for the ferromagnetic superconductors [S15]. However, a previous theoretical study [S16] suggests that, when a material is close to a ferromagnetic quantum critical point as in the case of UTe_2 [S6], the non-unitary pairing states can be stable even in a paramagnetic state. We note that, as stated in the main text, while the nature of magnetic fluctuations are still controversial, the ferromagnetic fluctuations may be related to the emergence of the $B_{3u}+iA_u$ state. Therefore, our results would promote further studies on the pairing mechanism.

V. SAMPLE CHARACTERIZATION

The shapes of crystals #A1, #B1, #C1, and #C2 are depicted in Figs. S4a-d, respectively. Crystals #A1, #B1, and #C1 have a cuboid shape with dimensions $455\times250\times95\,\mu\mathrm{m}^3$, $260\times155\times210\,\mu\mathrm{m}^3$, and $300\times250\times35\,\mu\mathrm{m}^3$, respectively, while crystal #C2 has a more complicated shape. As described later, because the $\Delta\lambda_i(T)$ analysis is based on a cuboid shape, we focus on the data of crystals #A1, #B1, and #C1 in the $\Delta\lambda_i(T)$ discussions

below. We note that the data of $\Delta f(T)$ providing the similar exponent values for all the crystals in the power-law fittings, $\Delta f(T) \propto T^n$, imply that qualitatively similar $\Delta\lambda_i(T)$ results with crystals #B1 and #C1 are also expected in crystal #C2 (Fig.S5).

Crystallographic orientations of the sample were determined using X-ray diffraction with the monochromated Mo-K_α radiation using an imaging plate diffractometer (R-AXIS-RAPID, Rigaku). An example of the diffraction image is shown in Fig. S6. Small bright area with a cross at its center corresponds to the blind area near the direct incident beam. Clear Bragg reflections are observed as black dots. The sharpness of the reflections ensures small mosaicity of the present crystal. All the reflections are successfully indexed using the UTe_2 lattice parameters, where some of the indices are shown. The small splitting observed in higher angles such as (3 -2 15) reflection is attributed to two narrowly separated incident X-ray beam, $K_{\alpha 1}$ and $K_{\alpha 2}$. No twin component were identifieds. From the diffraction data, the sample orientation relative to the sample stage is precisely obtained. Note also that unlike conventional back-reflection Laue photographs, present measurement with transmission geometry can examine fairly small samples used in the penetration depth measurements.

Figure S7a shows specific heat of crystal #C2 at zero field and under $\mu_0 H = 5\,\mathrm{T}$ along the a- and b-axes. The difference of T_c between $H \parallel a$ and $H \parallel b$ suggests a large anisotropy of the upper critical field as already demonstrated in previous studies. Figure S7b depicts the resistivity with the current direction of a-axis in crystal #R1 picked up from the same ampoule as crystals #C1 and #C2. The resistivity shows maximum around 50 K below which Kondo hybridization yields coherent electronic states. The resistivity at low T is shown in Fig. S7c. The onset of the superconducting transition temperature T_c^{on} is about 1.89 K, and the resistivity gets zero at $T_c^{\mathrm{zero}} \approx 1.77\,\mathrm{K}$. Compared with $T_c \approx 1.65\,\mathrm{K}$ obtained from a large change of $\Delta f(T)$ and C/T, T_c^{on} and T_c^{zero} are relatively high, and T_c^{on} is close to the onset of the small diamagnetic signal in $\Delta f(T)$ and the small jump of C/T (Fig. 2d). This indicates that a superconducting path exists between the electrodes even above $T_c \approx 1.65\,\mathrm{K}$.

VI. DERIVATION OF ANISOTROPIC PENETRATION DEPTH FROM FREQUENCY SHIFT

The frequency shift of the oscillator $\Delta f(T) \equiv f(T) - f(0)$ and ac magnetic susceptibility shift $\Delta\chi(T) \equiv \chi(T) - \chi(0)$ satisfy the relation [S17],

$$\frac{\Delta f(T)}{f_0} = -\frac{V_s}{2V_c(1-N)}\Delta\chi(T), \tag{S12}$$

where $f_0 = 13.8\,\text{MHz}$ is the resonant frequency without the sample, V_s and V_c are the sample and coil volumes, and N is the demagnetization factor calculated from the equation [S18]

$$\frac{1}{1-N} = 1 + \frac{4ab}{3c(a+b)}. \tag{S13}$$

$\Delta\chi_i$ data for crystals #A1, #B1, and #C1 with the magnetic field along i-axis calculated from the above equations are depicted in Figs. S8a-c, respectively. It is clearly seen that the values of $\Delta\chi_a(T_c)$ and $\Delta\chi_b(T_c)$ are nearly equal to 1, while $\Delta\chi_c(T_c)$ is much smaller than 1. This difference can be understood in consideration of the skin effect. The skin depth δ is calculated by

$$\delta = \sqrt{\frac{\rho}{\pi f_0 \mu_r \mu_0}}, \tag{S14}$$

where ρ is the resistivity, f is the frequency of the ac magnetic field, and μ_0 and μ_r are vacuum and relative permeability, respectively. For example, in the data of #C1, by substituting $\rho = 30\,\mu\Omega\text{cm}$ (Fig. S7c) and $\mu_r = 1$, we obtain $\delta = 75\,\mu\text{m}$. When the magnetic field is parallel to the a- or b-axes, the skin depth is larger than the sample thickness, and the skin effect can be neglected. Then, the total shift of $\Delta\chi_a(T_c) \approx \Delta\chi_b(T_c) \approx 1$ are expected in the perfect diamagnetic state. As shown in Fig. S6, $\Delta\chi_a(T_c) \approx \Delta\chi_b(T_c) \approx 1.05$ for crystal #C1 and 0.9 for crystal #B1, from which we can evaluate that the errors of the calculated N values are within 10%. On the other hand, when the magnetic field is parallel to the c-axis, the skin effect expels the magnetic field even in the normal state, which reduces the $\Delta\chi_c(T_c)$ value from unity as shown in Fig. S6. For crystal #C1, $\Delta\chi_c(T_c) \approx 0.8$ is expected for $\delta = 75\,\mu\text{m}$, which is consistent with the experimental value obtained from crystal #R1 picked up from the same batch as crystals #C1 and #C2. For crystal #A1 and #B1, we measured $\Delta\chi_i(T)$ up to 12 K and 9 K, respectively, (Fig. S8c). Since the skin depth becomes longer and compatible to the sample thickness with increasing temperature, $\Delta\chi_c$ reaches almost the same value of $\Delta\chi_a$ and $\Delta\chi_b$ at higher temperatures as shown in Fig. S6c. These

results indicate that our estimation of the demagnetization factors N is appropriate and $\Delta\chi(T)$ data are successfully derived from $\Delta f(T)$ data in crystals #A1, #B1, and #C1. We note that we measured $\Delta f(T)$ in another crystal (#C2) (see Fig. S5), but the complicated sample shape of crystal #C2 makes the quantitative analysis of $\Delta\lambda_i$ difficult. We have carried out the same analysis for crystal #C2 as for other crystals. However, the values of $\Delta\chi_{a,b}(T_c)$ for crystal #C2 are much larger than unity, indicating that the demagnetization factor N for crystal #C2 is not properly obtained probably because of the complicated sample shape. Hence, we did not carry out further analysis for crystal #C2.

In general, for a superconductor with anisotropic penetration depth components, χ_i can be expressed by using λ_j and λ_k through the equations [S19],

$$\begin{cases} 1+\chi_a = \dfrac{\lambda_b}{R_a^c} + \dfrac{\lambda_c}{R_a^b} & \text{(S15)} \\ 1+\chi_b = \dfrac{\lambda_c}{R_b^a} + \dfrac{\lambda_a}{R_b^c} & \text{(S16)} \\ 1+\chi_c = \dfrac{\lambda_a}{R_c^b} + \dfrac{\lambda_b}{R_c^a}, & \text{(S17)} \end{cases}$$

where R_i^j and R_i^k are the effective lengths when the magnetic field is applied along the i-axis.

The effective length R for a cylindrical shape with various aspect ratio is recently calculated in Ref. [S20]. Defining $R = \eta r$, where r is the radius of the cylinder, η as a function of the demagnetization factor N is approximately expressed as

$$\eta(N) = \frac{a + cN + eN^2}{1 + bN + dN^2}, \tag{S18}$$

where the coefficients are $a = 0.504$, $b = -0.517$, $c = -0.710$, $d = -0.446$, and $e = 0.206$. Then, in the case of rectangular shape, we define the effctive length as $R_k^i = 2\eta_k i$ with the η_k calculated by Eqs. (S13,S18) with the magnetic field along k-axis. We note that similar results can be obtained by using R proposed in the Ref. [S17]. Also, we stress that the robustness of our analyses against the errors of the effective dimensions has been confirmed by changing the values by $\pm 30\,\%$ in section VIII.

From the above discussions, we can derive $\Delta\lambda_a$, $\Delta\lambda_b$, and $\Delta\lambda_c$ through the relations,

$$R\Delta\lambda_i = -\frac{R_k^j R_i^k}{R_k^i}\Delta\chi_i + \frac{R_j^i R_k^j R_i^k}{R_k^i R_i^j}\Delta\chi_j + R_k^j \Delta\chi_k, \tag{S19}$$

where $R = 1 + R_b^a R_c^b R_a^c / R_b^c R_a^b R_c^a$. The calculated $\Delta\lambda_a/\lambda_a(0)$, $\Delta\lambda_b/\lambda_b(0)$, and $\Delta\lambda_c/\lambda_c(0)$ data for crystal #A1, #B1, and #C1 are shown in Fig. 4 in the main text.

VII. FITTING-RANGE DEPENDENCE OF EXPONENT VALUES AND POSSIBILITY OF DEEP GAP MINIMA

We checked the fitting range $T_{\rm max}$ dependence of the exponent values n_i in crystals #A1, #B1, and #C1 obtained by the power-law fitting, $\Delta\lambda_i \propto T^{n_i}$ (see Fig. S9). We confirmed that our discussions on the gap structure via n_i values are not affected by the $T_{\rm max}$ values. We have excluded the possibility of a line node gap in UTe_2 in section III. However, deep gap minima can be possible, for example, if c_2 and c_6 are much larger than the other coefficients in Eq. S5, but such a possibility can be ruled out by the following experimental facts. When the size of the gap minima is comparable or larger than the experimental lowest temperature, the exponent obtained from the power-law fitting becomes higher as the maximum value $T_{\rm max}$ of the fitting range gets lower [S21, S22]. In contrast, our experimental data do not show such a fitting-range dependence as shown in Fig. S9, indicating that the gap minima, if exist, should be really deep, much lower than the lowest temperature of our measurements. Even in the case of such deep minima, one can get important information from the sample dependence. In general, when the impurity scattering becomes large, the averaging effect of gap anisotropy leads to the increase of minimum gap, which results in a larger exponent value of low-temperature penetration depth. From the $T_{\rm c}$ values, we can consider that crystal #A1 is cleaner than crystals #B1 and #C1. However, we observed lower exponents in crystals #B1 and #C1 compared with those in crystal #A1 in the power-law analysis of penetration depth especially for the c-axis data, which is the opposite to the expected behavior of deep gap minima case. On the other hand, the observed tendency can be explained by the differenct sensitivity of the order parameters to the impurity scattering in our proposed chiral order parameter case as discussed in section IX.

VIII. ROBUSTNESS OF ANALYSES

It is known that the effective dimensions R_{3D} (Eq. S18) have usually errors of $\pm 20\,\%$ even in the isotropic case [S17]. Thus, we need to check the effect of the errors on our analyses, especially on the exponent values obtained by the power-law fitting. To this end, we varied the effective dimensions as $R_b^a \to r_{ab}R_b^a$, $R_c^a \to r_{ac}R_c^a$, $R_a^b \to r_{ba}R_a^b$, $R_c^b \to r_{bc}R_c^b$, $R_a^c \to r_{ca}R_a^c$, and $R_b^c \to r_{cb}R_b^c$ for all crystals. The r dependence of the exponent values n_i are summarized

in Fig. S10. We emphasize here that, while the n_a and n_b values in crystal #B1 are relatively sensitive to the r values, other exponent values and especially the anisotropy of the exponent n is quite stable againt r values. This result strongly confirms the conclusions in this study.

IX. INTERFERENCE EFFECT OF POINT NODES

In this section, we calculate $\Delta\lambda_i(T)/\lambda_i(0)$ with a simplified model for the $B_{3u}+iA_u$ state. We consider the order parameter

$$\mathbf{d} = \begin{pmatrix} k_x k_y k_z + i k_x \\ c_2 k_z + i k_y \\ c_3 k_y + i k_z \end{pmatrix}, \tag{S20}$$

which is obtained from Eq. S5 for $c_1 = c_4 = c_5 = c_6 = 1$. The positions of the point nodes are described by

$$\begin{cases} k_y = 0 \\ k_x = \pm\sqrt{c_2^2 - 1}k_z \end{cases} \quad \text{and} \quad \begin{cases} k_z = 0 \\ k_x = \pm\sqrt{c_3^2 - 1}k_y \end{cases}, \tag{S21}$$

when $c_2 > 1$ and $c_3 > 1$. Here, we define the positions of point nodes by the angles θ_n and ϕ_n where $\tan(\theta_n) = \sqrt{c_2^2 - 1}$ and $\tan(\phi_n) = \sqrt{c_3^2 - 1}$ (Figs. S11a,b). The superfluid density along the a-, b-, and c-axes are calculated from

$$\begin{cases} \rho_a^b = 1 - \dfrac{3}{4\pi T}\displaystyle\int_0^1 (1-z^2)\int_0^{2\pi}\begin{pmatrix}\cos^2(\phi) \\ \sin^2(\phi)\end{pmatrix}\int_0^\infty \cosh^{-2}\left(\dfrac{\sqrt{\epsilon^2 + \Delta_0^2(T)\hat{g}^2(\theta,\phi)}}{2T}\right)\mathrm{d}\epsilon\mathrm{d}\theta\mathrm{d}\phi \\ \rho_c = 1 - \dfrac{3}{4\pi T}\displaystyle\int_0^1 z^2\int_0^{2\pi}\int_0^\infty \cosh^{-2}\left(\dfrac{\sqrt{\epsilon^2 + \Delta_0^2(T)\hat{g}^2(\theta,\phi)}}{2T}\right)\mathrm{d}\epsilon\mathrm{d}\theta\mathrm{d}\phi \end{cases}, \tag{S22}$$

where $z = \cos(\theta)$, $\Delta_0(T)$ is the T dependence of the gap size, and $\hat{g}(\theta,\phi)$ is the angular dependence of the gap function with a maximum value of unity [S23]. In this study, we used an approximation [S24]

$$\Psi(t) = \frac{\pi T_c e^{-\langle\Omega^2 \ln|\Omega|\rangle}}{e^\gamma}\tanh\left(\mathrm{e}^\gamma\sqrt{\frac{8(1-\mathrm{t})}{7\zeta(3)\mathrm{t}}\frac{\mathrm{e}^{\langle\Omega^2\ln|\Omega|\rangle}}{\sqrt{\langle\Omega^4\rangle}}}\right) \tag{S23}$$

and $\Delta_0(T)\hat{g}(\theta,\phi) = \Psi(T)\Omega(\theta,\phi)$, where $\langle\Omega^2\rangle = 1$, $\gamma \approx 0.577$ is the Euler constant, and $\langle\cdots\rangle$ represents averaging over the Fermi surface. From $\rho_i(T)$, we can calculate the anisotropic magnetic penetration depth $\Delta\lambda_i(T)/\lambda(0) = 1/\sqrt{\rho_i(T)} - 1$.

First, we examine the nodal position dependence of $\Delta\lambda_i(T)$ in the $B_{3u} + iA_u$ state by changing the parameters c_2 and c_3 for the fixed parameters $c_1 = 0$, $c_4 = 1$, $c_5 = 1$, and $c_6 = 1$. As an example, the calculated $\Delta\lambda_i(T)/\lambda_i(0)$ for $\theta_n = 20^\circ$ and $\phi_n = 30^\circ$ are depicted in Fig. S12a. We obtained the exponent value n_c as a function of θ_n by fixing $\phi_n = 30^\circ$, which reflects the closeness of the nearby point nodes, by power-law fitting up to $0.3T_c$ (Fig. S12b). In this calculation, we find that the exponent value n_c approaches 1.6 as $\theta_n \to 0$, and $n_c < 2$ is robust when the node angle θ_n is less than 20°. These results can be interpreted as an interference effect due to nearby two point nodes, in which each point node can no longer be treated as an individual. Thus, the observation of $n_c < 2$ for crystals #B1 and #C1 is indicative of the closeness of two point nodes.

Next, we investigate the temperature dependent exponent values by changing the parameters of $B_{3u} + iA_u$ state in Eq. S5. The gap structures change as shown in Fig. S13 when the c_4 value is varied (here, we fixed $c_1 = 0$, $c_5 = 1$, $c_6 = 1$, $c_2 = \sqrt{c_6^2 + c_4^2/\tanh^2(\pi - \theta_n)}$, and $c_3 = \sqrt{c_5^2 + c_4^2/\tanh^2(\pi - \phi_n)}$ for $\theta_n = 15^\circ$ and $\phi_n = 30^\circ$). The temperature dependence of the exponents $n_i(T/T_c)$ can be calculated from

$$n_i = \frac{d\left[\ln\left(\Delta\lambda_i(T/T_c)\right)\right]}{d\left[\ln\left(T/T_c\right)\right]} \tag{S24}$$

and the results are summarized in Figs. S14 and S15. We find that for all the parameters we use, n_c is smaller than 2 in the temperature range above $\sim 0.1T_c$, where we can analyze our experimental data, whereas it starts approaching 2 below $\sim 0.1T_c$ except for $n_c \sim 1.5$ at $\theta_n = 0^\circ$ (Fig. S14b) where the dispersion of the gap function near the point nodes is not linear but quadratic (Fig. S13d). This low-temperature recovery of the power of 2 is simply because the nearby point nodes can be treated as individual nodes in the $T \to 0$ limit. The crossover energy from T^2 to lower exponent region is determined by the maximum gap size between the nearby point nodes, and thus it depends on the locations of nodes and gap sizes of the B_{3u} and A_u components.

In order to understand the sample dependence of the n_c value, we compare the results of crystals #A1, #B1, and #C1 with different T_cs. We find a trend that the n_c value approachs 2 as T_c increases, which can be explained based on the chiral $B_{3u} + iA_u$ state as follows. As stated in the main text, we assume here that the B_{3u} component is more suppressed by the nonmagnetic impurity scattering than the A_u component. Then, the A_u component become more dominant in lower-T_c samples. Considering the qualitative correspondence between

relative size of the two order parameters and the position of point nodes (Table II), as the A_u component becomes dominant, the point nodes get closer to each other near the k_z-axis. Thus, we can expect that the interference effect of the point nodes tend to change the exponent values in the lower-T_c samples. Furthermore, the above picture is also consistent with the larger $\Delta\lambda_c(T)/\lambda_c(0)$ in the lower-T_c samples than in the higher-T_c sample. We note that, while the impurity effect on the chiral superconducting state is complicated, the expectation that the B_{3u} component is more sensitive than the A_u component is natural because the B_{3u} state is solely more anisotropic than the A_u state.

We comment on the calculated n_a and n_b values which are slightly different from the observed $n_a \approx n_b \approx 2$. The reason for this difference may be related to the FS geometry and the gap size. As mentioned in the main text, while the quantum oscillation measurements show quasi-two-dimensional (2D) FSs, the recent ARPES study [S25] shows a 3D FS around the Z point and quasi-2D FSs. Then, we can expect that $\Delta\lambda_a(T)/\lambda_a(0)$ and $\Delta\lambda_b(T)/\lambda_b(0)$ are mainly contributed from the point nodes on the quasi-2D FSs, while $\Delta\lambda_c(T)/\lambda_c(0)$ is mainly contributed from the point nodes on the 3D FS. Therefore, our calculations based on a spherical FS lead to the calculated n_a and n_b values different from the experimental values. On the other hand, the exponent values in the low-temperature limit are stable against the FS geometries and the gap size because they are determined only by the shape of nodes and the energy dispersion near the nodes. For example, point nodes with a linear dispersion give the exponent value of 2, which is consistent with Fig. S14 and S15 in the low-temperature limit. In particular, the specific heat jump at T_c is larger than the weak-coupling value, suggesting the strong coupling nature in UTe_2. In such cases, the thermal energy with respect to the gap size is smaller in the experiments than in the calculations. Therefore, we should compare the experimental exponents with the calculated ones in the low-temperature region. Figure S14 and S15 show the exponent values near 2 in the low-temperature region when the interference effects of point nodes are negligible, which is consistent with the experimental values obtained in the ultraclean sample (#A1).

X. ESTIMATION OF $\lambda(0)$

The $\lambda(0)$ value can be usually estimated from the lower critical field $H_{\rm c1}$ through the equation, $\mu_0 H_{\rm c1} = (\phi_0/4\pi\lambda^2)(\ln\kappa + 0.5)$, where ϕ_0 is the magnetic flux quantum and κ is the Ginzburg-Landau (GL) parameter. The $H_{\rm c1}$ measurements are conducted in Ref. [S26], showing an anomalous anisotropic enhancement of $H_{\rm c1}$ for $H \parallel b$ and $H \parallel c$. This result indicates unusual additional contributions to the vortex line energy possibly related to the Ising-like ferromagnetic fluctuations. While the origin of this enhancement is not established, it is obvious that these anomalous contributions make the estimation of $\lambda(0)$ from the $H_{\rm c1}$ value quite challenging. Therefore, to quantify the anisotropy of the quasiparticle excitations, we need to estimate the anisotropy of $\lambda(0)$ from another perspective.

Alternatively, we estimate the anisotropy in $\lambda_i(0)$ from the anisotropy in $\xi_i(T_{\rm c})$. The anisotropy in $\xi_i(T_{\rm c})$ is calculated from the initial slope in $\mu_0 H_{c2}(T)$ obtained from specific heat measurements on an ultra-clean sample picked up from the same batch with the sample used in the de-Haas van-Alphen measurement in Ref. [S27] [S26]. In general, however, these anisotropies are not the same in superconductors with an anisotropic gap structure. The anisotropies of $\lambda(0)$ and $\xi(T_c)$ can be given by $\gamma_\lambda^2(0) = \lambda_j^2(0)/\lambda_i^2(0) = \langle v_{F,i}^2\rangle/\langle v_{F,j}^2\rangle$ and $\gamma_\xi^2(T_c) = \xi_i^2(T_c)/\xi_j^2(T_c) = \langle\Omega^2 v_{F,i}^2\rangle/\langle\Omega^2 v_{F,j}^2\rangle$, respectively, where Ω describes the angular dependence of the gap function [S28]. Thus, the anisotropy of $\lambda(0)$ cannot be estimated from the anisotropy of $\xi(T_c)$ when the superconducting gap function has a strong momentum dependence. However, the correction is not that large: In the recent calculations for the gap structure with two point nodes (like the B_{3u} state) on a spherical Fermi surface, the ratio of $\gamma_\xi(T_c)$ to $\gamma_\lambda(0)$ is as small as $\sqrt{2}$ [S24], which does not change the main results that the a-axis quasiparticle excitations are significantly small compared with those along other directions (see Fig. 4).

In this study, therefore, for simplicity we ignored the anisotropy of gap function for the relation between the anisotropies of coherence length and penetration depth. As a result, we obtained $\lambda_a(0) \sim \lambda_c(0) > \lambda_b(0)$, which is consistent with the band structure calculations that the bands along the a- and c-axes consist of the d- and f-orbitals of U, while the band along the b-axis consists of the p-orbitals of Te (see e.g., Refs. [S12, S13, S16, S29, S30]). Note that this does not affect the exponent analysis of the low-temperature penetration depth.

If we consider the effect of gap anisotropy Ω in the proposed $B_{3u} + iA_u$ case, we expect that the ratio $\left\langle \Omega^2 v_{F,i}^2 \right\rangle / \left\langle v_{F,i}^2 \right\rangle$ is largest for a-axis, near which nodes are absent. This immediately implies that in comparison with the isotropic approximation we use, the actual ratio $\xi_i^2(T_c)/\lambda_i^2(0)$ should be larger for a-axis, and thus the $\lambda_a(0)$ value is expected to be even larger. Then, the a-axis data $\Delta\lambda_a(T)/\lambda_a(0)$ should be even smaller, and thus the anisotropy of low-energy quasiparticle excitations becomes more pronounced. From these reasons, we argue that the results of this simple analysis are meaningful, and the conclusions obtained are valid.

Table S1. **Basis functions and nodal types for even-parity order parameters in the point group** D_{2h}**.**

IR	Basis functions	Nodes
A_{1g}	k_x^2, k_y^2, k_z^2	None
B_{1g}	$k_x k_y$	Line
B_{2g}	$k_z k_x$	Line
B_{3g}	$k_x k_y$	Line

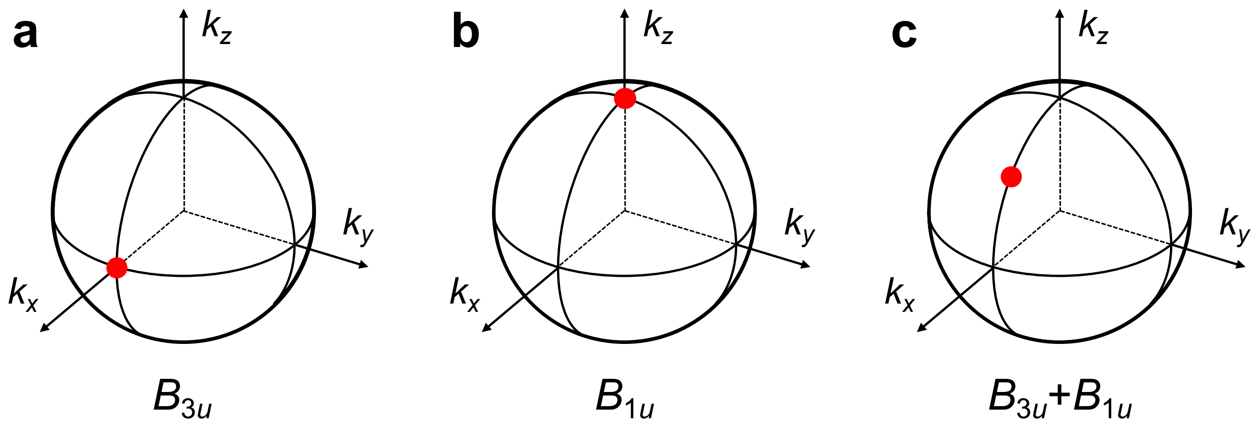

FIG. S1. **Nodal positions induced by B_{3u} and B_{1u} states preserving time-reversal symmetry. a-c,** Positions of the point nodes for the B_{3u} (**a**), B_{1u} (**b**), and $B_{3u} + B_{1u}$ states (**c**).

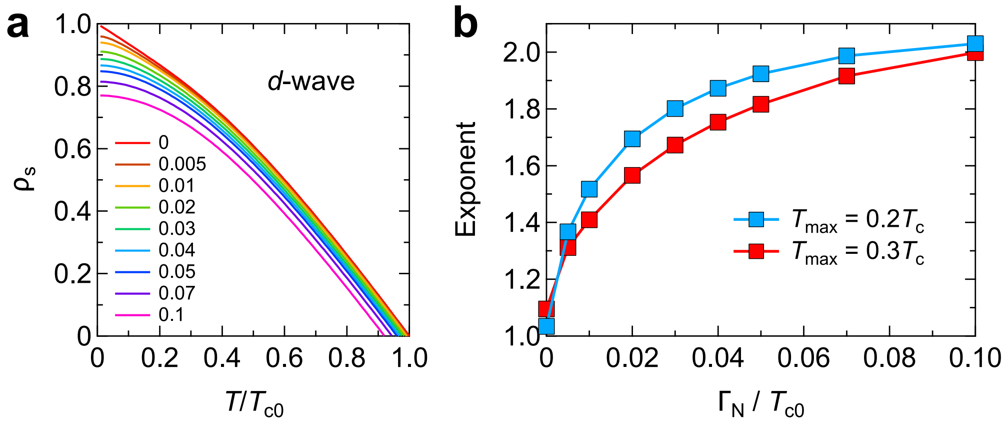

FIG. S2. **Superfluid density in dirty d-wave superconductors. a,** Superfluid density as a function of the normalized temperature calculated with various Γ_N/T_{c0} values in the unitary limit. **b,** The exponent values obtained from the power-law fitting to the superfluid density. T_{max} is the upper bound of the fitting range.

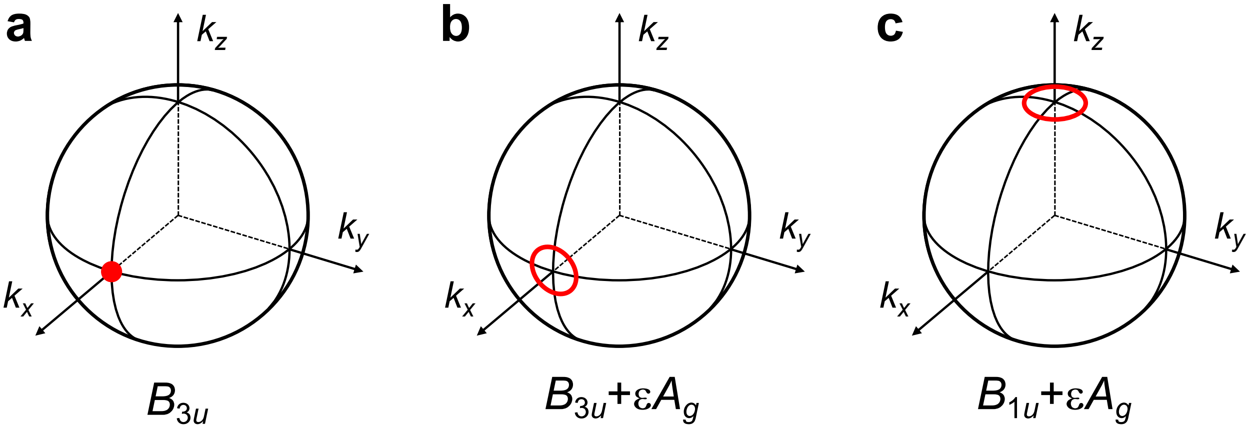

FIG. S3. **Nodal positions induced by B_{3u} and A_u states preserving time-reversal symmetry. a-c,** Positions of the nodes for the B_{3u} (**a**), $B_{3u} + \varepsilon A_g$ (**b**), and $B_{1u} + \varepsilon A_g$ states (**c**), where ε is a small real number.

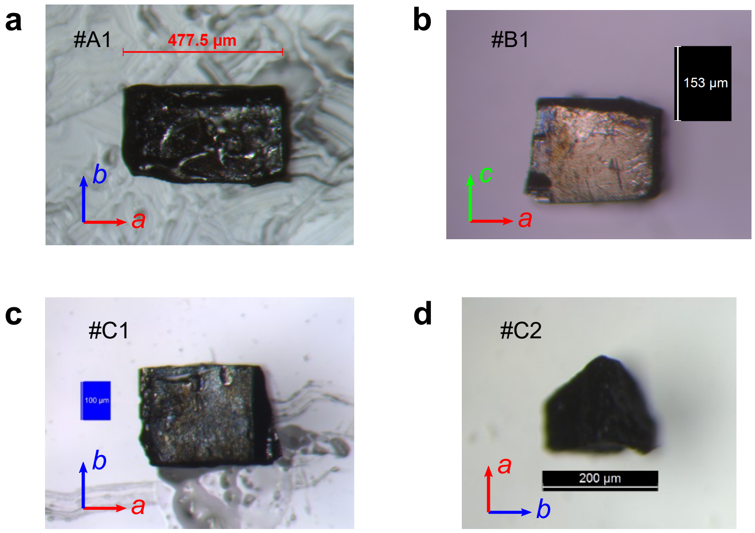

FIG. S4. **Geometry of the UTe_2 single crystals. a-d,** Pictures of single crystals #A1 (**a**), #B1 (**b**), #C1 (**c**), and #C2 (**d**).

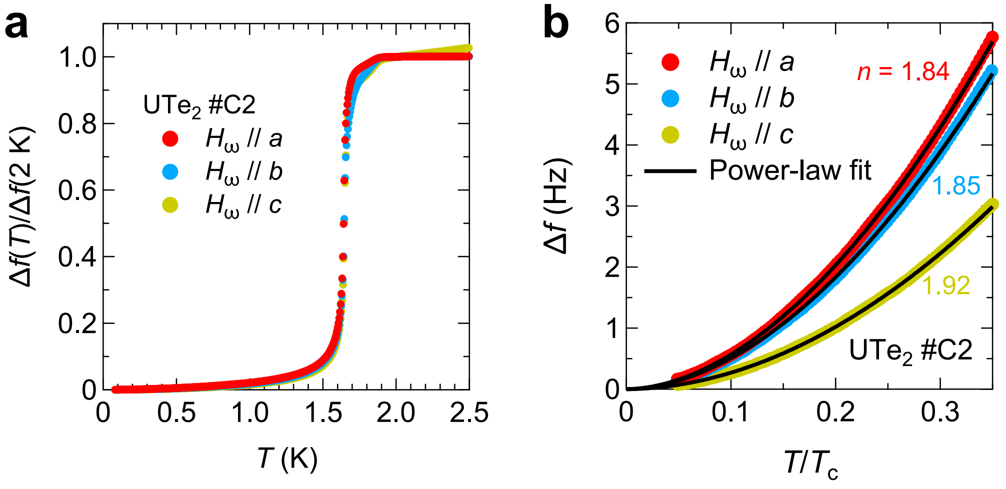

FIG. S5. **Frequency shift for crystal #C2. a,** Overall temperature dependence of the frequency shift normalized by the value at 2 K with the magnetic field along each crystallographic axis. **b**, Low-T behavior of Δf for crystal #C2. The black lines are fitting curves with the power-law finction.

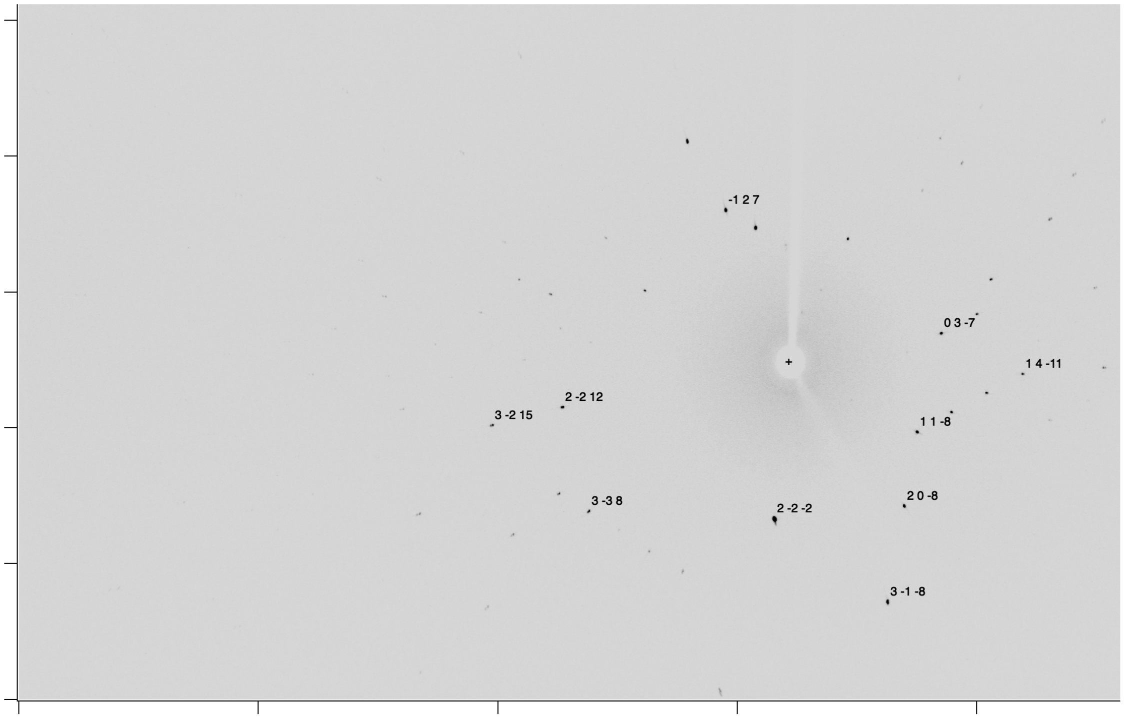

FIG. S6. **Diffraction image of XRD measurement.** An example of the diffraction image of XRD measurement. Some reflection indices of the spots are shown in the figure.

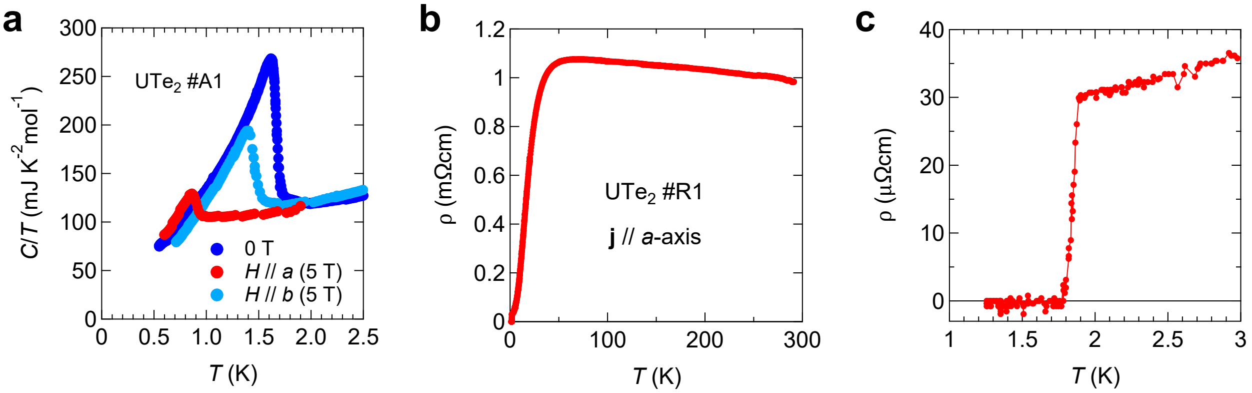

FIG. S7. **Specific heat and resistivity. a,** Specific heat of crystal #C1 at zero field and under 5 T along the a- and b-axes. **b,c,** The resistivity of crystal #R1 with the current along the a-axis in whole T range (**b**) and at low T (**c**).

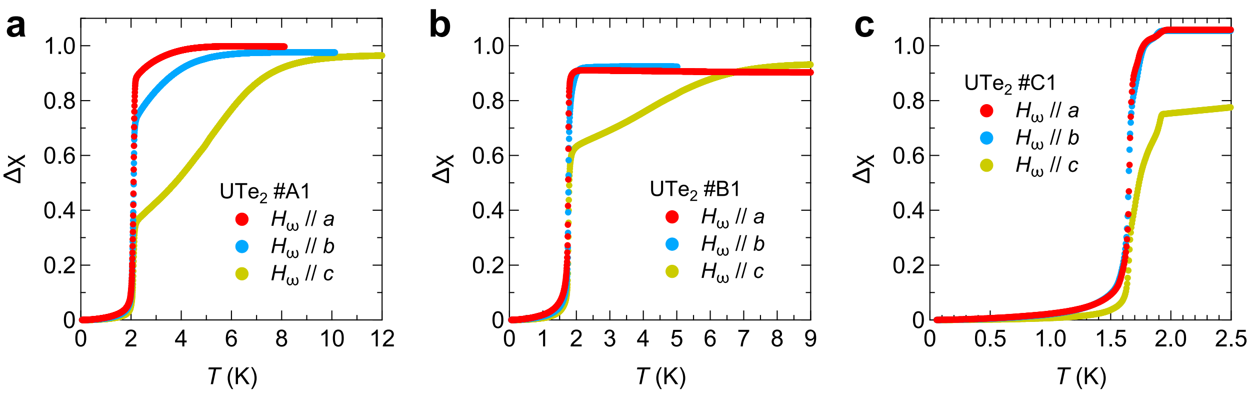

FIG. S8. **Magnetic susceptibility along three crystallographic axes. a-c,** Magnetic susceptibility shift $\Delta\chi \equiv \chi(T) - \chi(0)$ calculated from Δf through Eq. S12 for crystals #A1 (**a**), #B1 (**b**), and #C1 (**c**).

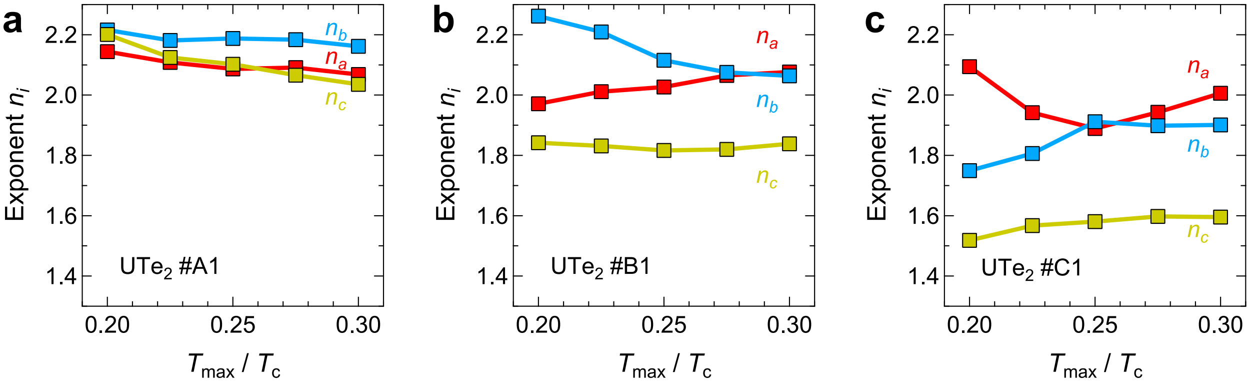

FIG. S9. **Fitting-range dependence of the exponent values. a-c,** Exponent values n_i for crystals #A1 (**a**), #B1 (**b**), and #C1 (**c**) as a function of the maximum value of the fitting range T_{max}.

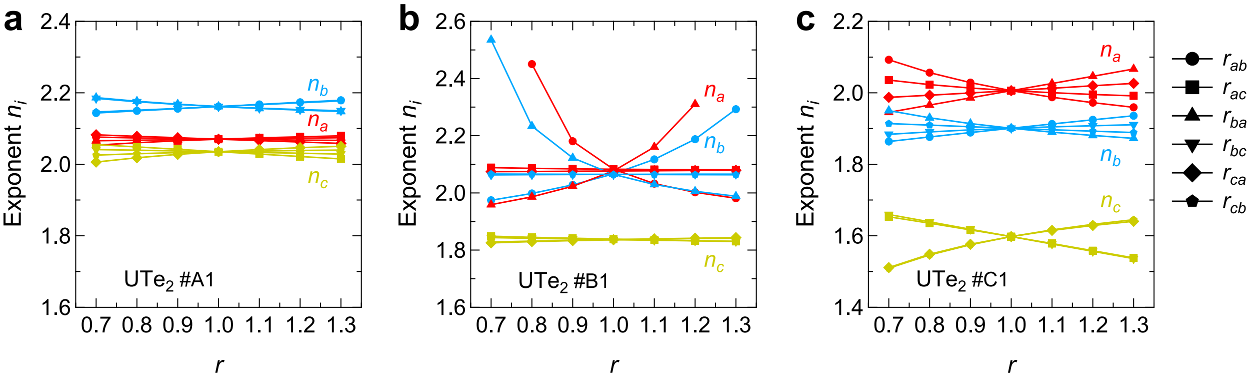

FIG. S10. **Effective length dependence of the exponent values. a-c,** Exponent values obtained from the power-law fitting to $\Delta\lambda_i(T)$ as a function of r in crystals #A1 (**a**), #B1 (**b**), and #C1 (**c**).

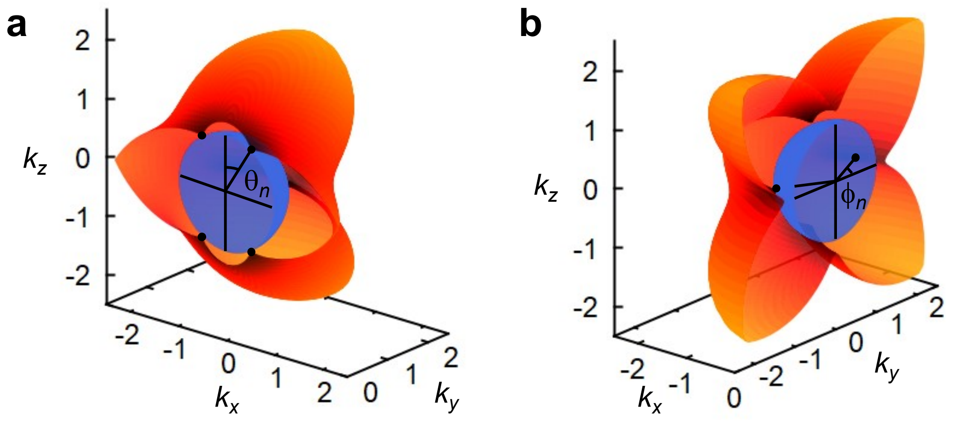

FIG. S11. **Gap structure of the $B_{3u} + iA_u$ state. a,b,** Angular dependence of the $B_{3u} + iA_u$ gap function and definitions of θ_n (**a**) and ϕ_n (**b**), respectively.

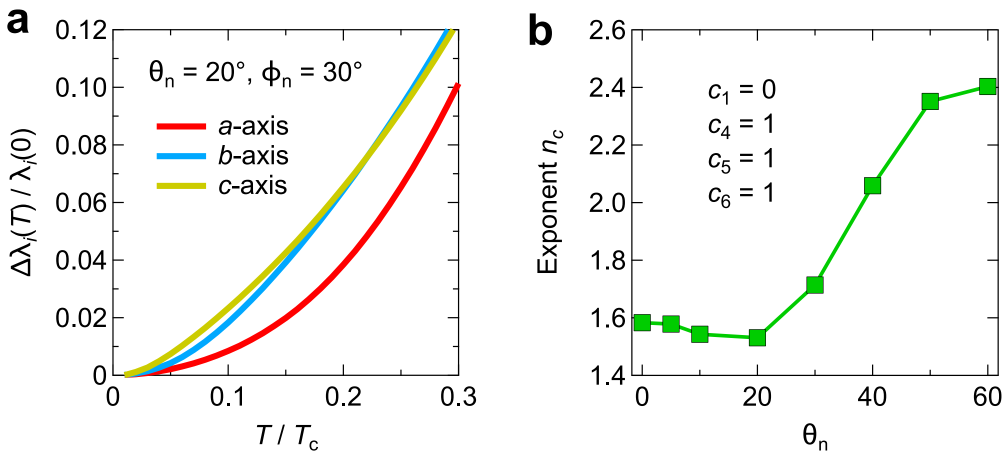

FIG. S12. **Calculated anisotropic penetration depth and exponent values. a,** Calculated $\Delta\lambda_i(T)/\lambda_i(0)$ along the $i = a$, b, and c axes with point nodes in the $\theta_n = 20°$ and $\phi_n = 30°$ directions. **b,** Exponent values obtained from the power-law fitting $\Delta\lambda_c(T)/\lambda_c(0) \propto T^{n_c}$ as a function of θ_n with the fixed value of $\phi_n = 30°$.

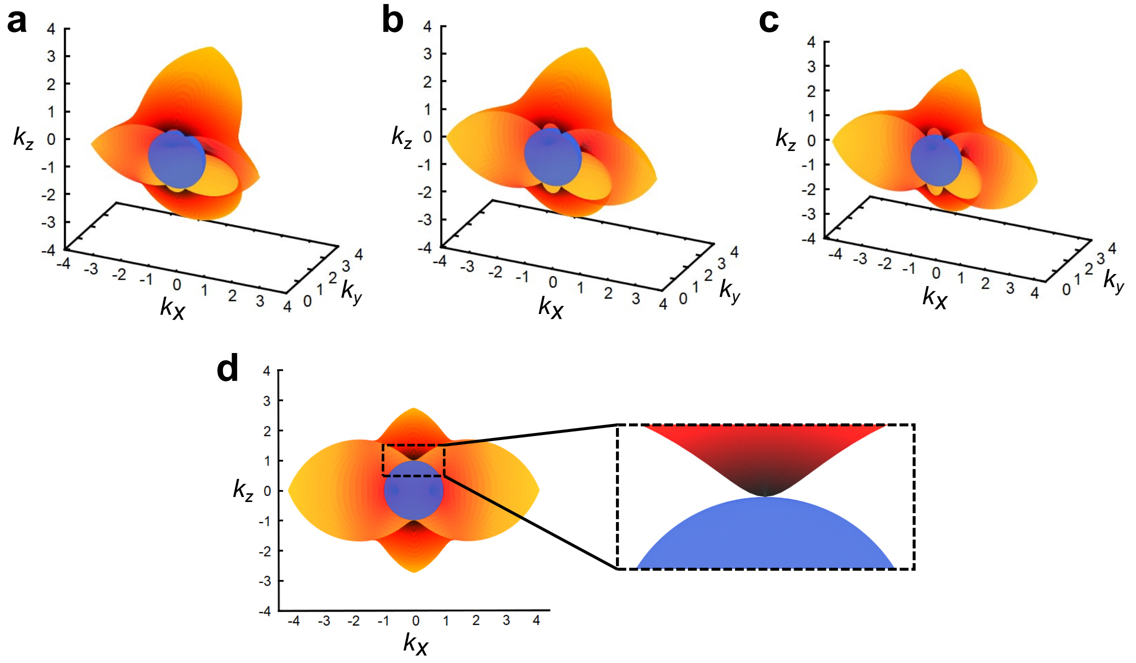

FIG. S13. c_4 **value dependence of the gap structure in the** $B_{3u} + iA_u$ **state. a-c,** Angular dependence of the $B_{3u} + iA_u$ gap function with $c_4 = 1.0$ (**a**), $c_4 = 1.5$ (**b**), and $c_4 = 2.0$ (**c,d**). The other parameters are fixed as $c_1 = 0$, $c_5 = 1$, $c_6 = 1$, $c_2 = \sqrt{c_6^2 + c_4^2/\tanh^2(\pi - \theta_n)}$, and $c_3 = \sqrt{c_5^2 + c_4^2/\tanh^2(\pi - \phi_n)}$ for $\theta_n = 15°$ and $\phi_n = 30°$. **d,** The same calculation with $c_4 = 2.0$ and $\theta_n = 0$.

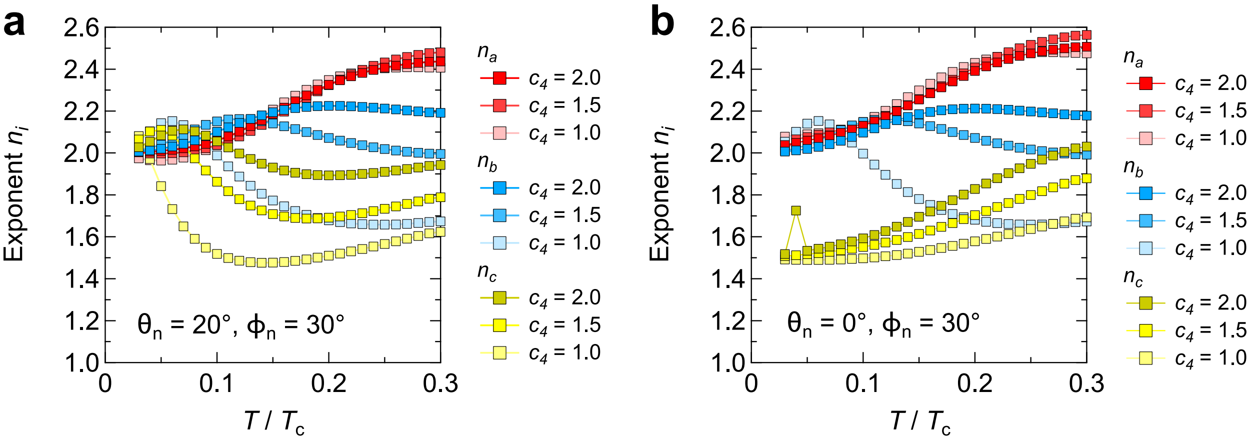

FIG. S14. **Temperature dependent exponent values in the** $B_{3u} + iA_u$ **state. a,b,** Temperature dependent exponents obtained by Eq. S24 by varying the c_4 value while fixing the angles $\theta_n = 20°$ and $\phi_n = 30°$ (**a**) and $\theta_n = 0°$ and $\phi_n = 30°$ (**b**).

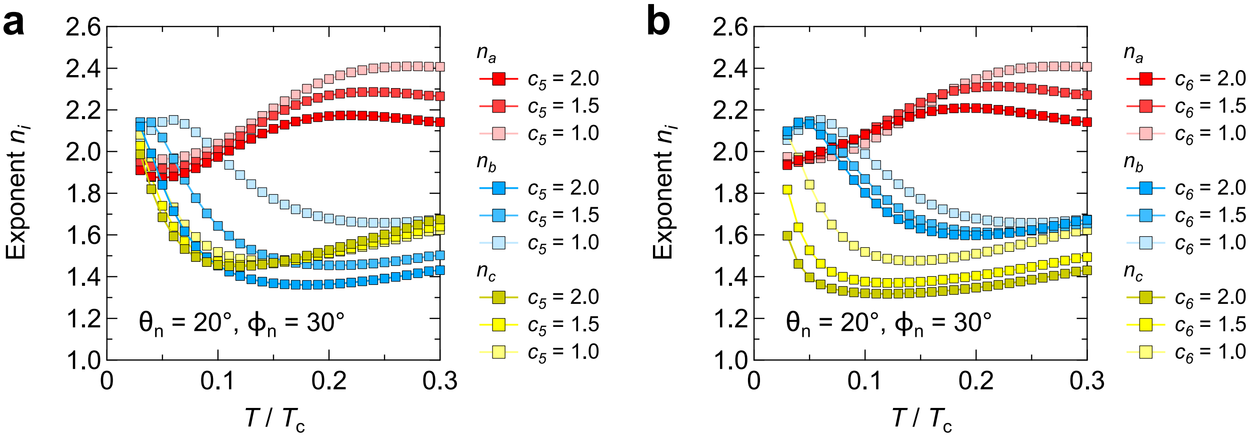

FIG. S15. **c_5 and c_6 dependence of the exponent values. a,b,** Temperature dependent exponents obtained by Eq. S24 by varying the c_5 and c_6 value while fixing the angles $\theta_n = 20°$ and $\phi_n = 30°$.

SUPPLEMENTARY REFERENCES

[S1] Hayes, I. M. *et al.* Multicomponent superconducting order parameter in UTe_2. *Science* **373**, 797–801 (2021). URL https://www.science.org/doi/abs/10.1126/science.abb0272.

[S2] Hirschfeld, P. J. & Goldenfeld, N. Effect of strong scattering on the low-temperaturepenetration depth of a d-wave superconductor. *Phys. Rev. B* **48**, 4219–4222 (1993). URL https://link.aps.org/doi/10.1103/PhysRevB.48.4219.

[S3] Hashimoto, K. *et al.* Anomalous superfluid density in quantum critical superconductors. *Proc. Natl. Acad. Sci. USA* **110**, 3293–3297 (2013). URL https://www.pnas.org/content/110/9/3293.

[S4] Mizukami, Y. *et al.* Evolution of quasiparticle excitations with enhanced electron correlations in superconducting $A\mathrm{Fe_2As_2}$ (A = K, Rb, and Cs). *Phys. Rev. B* **94**, 024508 (2016). URL https://link.aps.org/doi/10.1103/PhysRevB.94.024508.

[S5] Kosztin, I. & Leggett, A. J. Nonlocal effects on the magnetic penetration depth in d-wave superconductors. *Phys. Rev. Lett.* **79**, 135–138 (1997). URL https://link.aps.org/doi/10.1103/PhysRevLett.79.135.

[S6] Ran, S. *et al.* Nearly ferromagnetic spin-triplet superconductivity. *Science* **365**, 684–687 (2019). URL https://science.sciencemag.org/content/365/6454/684.

[S7] Sakai, H. *et al.* Single crystal growth of superconducting UTe_2 by molten salt flux method. *Phys. Rev. Materials* **6**, 073401 (2022). URL https://link.aps.org/doi/10.1103/PhysRevMaterials.6.073401.

[S8] Hirschfeld, P. J. *et al.* Consequences of resonant impurity scattering in anisotropic superconductors: Thermal and spin relaxation properties. *Phys. Rev. B* **37**, 83 (1988). URL https://journals.aps.org/prb/abstract/10.1103/PhysRevB.37.83.

[S9] Bae, S. *et al.* Anomalous normal fluid response in a chiral superconductor UTe_2. *Nat. Commun.* **12**, 2644 (2021). URL https://doi.org/10.1038/s41467-021-22906-6.

[S10] Groß, F. *et al.* Anomalous temperature dependence of the magnetic field penetration depth in superconducting UBe_{13}. *Z. Phys. B* **64**, 175–188 (1986).

[S11] Kang, B. *et al.* Orbital selective Kondo effect in heavy fermion superconductor

UTe_2. *npj Quantum Mater.* **7**, 64 (2022). URL https://www.nature.com/articles/s41535-022-00469-z.

[S12] Xu, Y. *et al.* Quasi-Two-Dimensional Fermi Surfaces and Unitary Spin-Triplet Pairing in the Heavy Fermion Superconductor UTe_2. *Phys. Rev. Lett.* **123**, 217002 (2019). URL https://journals.aps.org/prl/abstract/10.1103/PhysRevLett.123.217002.

[S13] Ishizuka, J. & Yanase, Y. Periodic anderson model for magnetism and superconductivity in UTe_2. *Phys. Rev. B* **103**, 094504 (2021). URL https://link.aps.org/doi/10.1103/PhysRevB.103.094504.

[S14] Sigrist, M. & Ueda, K. Phenomenological theory of unconventional superconductivity. *Rev. Mod. Phys.* **63**, 239–311(1991). URL https://link.aps.org/doi/10.1103/RevModPhys.63.239.

[S15] Aoki, D., Ishida, K. & Flouquet, J. Review of U-based ferromagnetic superconductors: Comparison between UGe_2, URhGe, and UCoGe. *J. Phys. Soc. Jpn.* **88**, 022001 (2019). URL https://doi.org/10.7566/JPSJ.88.022001.

[S16] Nevidomskyy, A. H. Stability of a nonunitary triplet pairing on the border of magnetism in UTe_2. *preprint* arXiv:2001.02699 (2020).

[S17] Prozorov, R., Giannetta, R. W., Carrington, A. & Araujo-Moreira, F. M. Meissner-london state in superconductors of rectangular cross section in a perpendicular magnetic field. *Phys. Rev. B* **62**, 115–118 (2000). URL https://link.aps.org/doi/10.1103/PhysRevB.62.115.

[S18] Prozorov, R. & Kogan, V. G. Effective demagnetizing factors of diamagnetic samples of various shapes. *Phys. Rev. Applied* **10**, 014030 (2018). URL https://link.aps.org/doi/10.1103/PhysRevApplied.10.014030.

[S19] Prozorov, R. Meissner-London state in anisotropic superconductors of cuboidal shape. *preprint* arXiv:2101.06489 (2021).

[S20] Prozorov, R. Meissner-london susceptibility of superconducting right circular cylinders in an axial magnetic field. *Phys. Rev. Applied* **16**, 024014 (2021). URL https://link.aps.org/doi/10.1103/PhysRevApplied.16.024014.

[S21] Cho, K. *et al.* Energy gap evolution across the superconductivity dome in single crystals of $(Ba_{1-x}K_x)Fe_2As_2$. *Sci. Adv.* **2**, e1600807 (2016). URL https://link.aps.org/doi/10.1126/sciadv.1600807.

[S22] Ishihara, K. *et al.* Tuning the parity mixing of singlet-septet pairing in a half-Heusler super-

conductor. *Phys. Rev. X* **11**, 041048 (2021). URL https://link.aps.org/doi/10.1103/PhysRevApplied.16.024014.

[S23] Prozorov, R. & Giannetta, R. W. Magnetic penetration depth in unconventional superconductors. *Supercond. Sci. Technol.* **19**, R41–R67 (2006). URL https://doi.org/10.1088/0953-2048/19/8/r01.

[S24] Kogan, V. G. & Prozorov, R. Temperature dependence of London penetration depth anisotropy in superconductors with anisotropic order parameters. *Phys. Rev. B* **103**, 054502 (2021). URL https://link.aps.org/doi/10.1103/PhysRevB.103.054502.

[S25] Miao, L. *et al.* Low energy band structure and symmetries of UTe_2 from angle-resolved photoemission spectroscopy. *Phys. Rev. Lett.***124**, 076401 (2020). URL https://link.aps.org/doi/10.1103/PhysRevLett.124.076401.

[S26] Ishihara, K. Anisotropic enhancement of lower critical field in ultraclean crystals of spin-triplet superconductor candidate UTe_2. *Phys. Rev. Research* **5**, L022002 (2023). URL https://link.aps.org/doi/10.1103/PhysRevResearch.5.L022002.

[S27] Aoki, D. *et al.* First Observation of the de Haas-van Alphen Effect and Fermi Surfaces in the Unconventional Superconductor UTe_2. *J. Phys. Soc. Jpn.* **91**, 083704 (2022). URL https://doi.org/10.7566/JPSJ.91.083704.

[S28] Kogan, V. G. , Prozorov, R. & Koshelev, A. E. Temperature-dependent anisotropies of upper critical field and London penetration depth. *Phys. Rev. B* **100**, 014518 (2019). URL https://link.aps.org/doi/10.1103/PhysRevB.100.014518.

[S29] Shishidou, T., Suh, H. G., Brydon, P. M. R., Weinert, M. & Agterberg, D. F. Topological band and superconductivity in UTe_2. *Phys. Rev. B* **103**, 104504 (2021). URL https://link.aps.org/doi/10.1103/PhysRevB.103.104504.

[S30] Ishizuka, J., Sumita, S., Daido, A. & Yanase, Y. Insulator-metal transition and topological superconductivity in UTe_2 from a first-principles calculation. *Phys. Rev. Lett.* **123**, 217001 (2019). URL https://link.aps.org/doi/10.1103/PhysRevLett.123.217001.
